# Association Between Diabetic Retinopathy and Periodontitis—A Systematic Review

**DOI:** 10.3389/fpubh.2020.550614

**Published:** 2021-01-08

**Authors:** María Olimpia Paz Alvarenga, Giza Hellen Nonato Miranda, Railson Oliveira Ferreira, Miki Taketomi Saito, Nathália Carolina Fernandes Fagundes, Lucianne Cople Maia, Rafael Rodrigues Lima

**Affiliations:** ^1^Laboratory of Functional and Structural Biology, Institute of Biological Sciences, Federal University of Pará, Belém, Brazil; ^2^Faculty of Dentistry, Institute of Health Sciences, Federal University of Pará, Belém, Brazil; ^3^Faculty of Medicine and Dentistry, School of Dentistry, University of Alberta, Edmonton, AB, Canada; ^4^Department of Pediatric Dentistry and Orthodontics, School of Dentistry, Federal University of Rio de Janeiro, Rio de Janeiro, Brazil

**Keywords:** diabetic retinopathy, periodontal disease, diabetes mellitus, systematic review, periodontitis (PD)

## Abstract

**Background:** Diabetic retinopathy is a common microvascular complication in diabetic patients and is considered the main cause of visual loss worldwide. Periodontitis is a chronic inflammatory condition, which compromises dental supporting tissues. The chronic bacterial challenge in periodontitis is a persistent source of inflammatory mediators that may be associated with insulin resistance, increasing the risk of complications of diabetes mellitus. This systematic review aimed to summarize the evidence in the association between diabetic retinopathy and periodontitis.

**Methods:** This review was registered under the number CRD 42019142267. A search strategy in five electronic databases and a gray literature source was performed based on the PECO acronym. After data extraction, the qualitative synthesis and risk of bias analyses were performed using the Newcastle–Ottawa scale. The level of evidence of all studies taken together was evaluated through the Grading of Recommendations Assessment, Development, and Evaluation (GRADE) approach.

**Results:** Out of the 253 citations screened, five cross-sectional studies met the eligibility criteria and were included in the qualitative analysis, in which two were judged to be of good quality, one as fair quality, and two as poor quality. Among the included studies, a significant relationship between the severity of periodontitis (CAL > 5 mm) and the severity of diabetic retinopathy (*p* < *0.05*) was reported by four studies. Also, an association between both diseases in non-obese adults was found after adjustments [OR 2.206 (1.114–4.366); *p* = 0.0232). However, the analysis of evidence by GRADE assessment was rated as low.

**Conclusions:** Although the results of individual studies suggest an association between diabetic retinopathy and periodontitis, the quality of the body of evidence was judged to be low by the GRADE approach. Further studies with larger sample sizes, adequate models of cofounders' adjustments, and prospective analysis of periodontitis and diabetes conditions ought to be conducted to clarify this association.

## Introduction

Diabetic retinopathy is a common microvascular complication in individuals with diabetes mellitus caused by prolonged hyperglycemia (1). Retinal vascularization disorders may affect about 37% of patients with diabetes (2), an alarming rate considering that The International Diabetes Federation estimates ~425 million adults had diabetes in 2017, turning diabetic retinopathy one of the most important causes of vision loss worldwide (3). Retinopathy in diabetic patients is caused by changes in retinal microvasculature after prolonged exposure to high blood glucose due to a lack of long-term diabetes control (1, 2, 4). Several biochemical mechanisms have been proposed that promote this vascular disruption such as oxidative stress and inflammation (5).

Periodontitis is a chronic inflammatory condition with high prevalence in diabetic patients, in which protective structures and dental support are compromised (6). According to the US National Health and Nutrition Examination Survey (NHANES) III, patients with glycated hemoglobin (HbA1c) levels >9% have a higher prevalence of severe periodontitis when compared to non-diabetic patients. Bidirectional interference has been also reported (7). The chronic bacterial challenge resulting from periodontitis is a persistent source of inflammatory mediators (8) that might interfere directly and indirectly in several systems (9, 10) and promote effects in diabetes as insulin resistance, being able to increase the onset of diabetes complications (7, 11).

Thus, this systematic review sought to gather studies that evaluated the presence of diabetic retinopathy in diabetic patients with and without periodontitis and analyzed the body of the evidence to know whether there exists a significant association between these diseases.

## Methods

### Protocol and Registration

The following systematic review was registered in the Prospective International Registry of Systematic Reviews—PROSPERO 2019 (http://www.crd.york.ac.uk) under the number CRD 42019142267, and it was developed based on the PRISMA (Preferred Reporting Items for Systematic Review and Meta-Analysis) protocol for systematic reviews (http://www.prisma-statement.org). A PRISMA checklist was performed as shown in Supplementary Table 1.

Studies were selected based on the PECO question, including observational studies in humans with diabetes mellitus (P—persons) in which periodontitis was present (E—exposure) or absent (C—comparison) to observe an association between this and diabetic retinopathy (O—outcome). Hence, we aimed to answer the focus question: Is there any association between periodontitis and diabetic retinopathy?

### Eligibility Criteria

The exclusion criteria applied in the full-text analysis were studies including patients under 12 years old, smokers, and other comorbidities in addition to diabetic retinopathy and diabetes mellitus, studies with groups evaluating gingivitis only, studies with no control group, case reports, descriptive studies, review articles, opinion articles, technical articles, guidelines, studies on animals, and *in vitro* experiments. No restrictions were placed on publication date nor language used.

The primary diagnostic criteria of periodontitis taken into consideration in this review were: clinical attachment loss (CAL)/probing depth >3 mm, bleeding on probing (BOP% > 25%) of evaluated sites, and/or >30% of radiographic bone loss. A secondary diagnostic criterion considered for periodontitis evaluation was community periodontal index (CPI) score 3 (probing depth of 3.5–5.5 mm) and score 4 (probing depth > 5.5 mm).

### Search Strategy

Five electronic databases—PubMed, Scopus, Web of Science, The Cochrane Library, and LILACS, and a gray literature source—OpenGrey were accessed to perform the search strategy from August 2019 to September 2020. The MeSH terms used were “Diabetes Mellitus,” “Adult,” “Periodontitis,” “Periodontal Diseases,” “Alveolar Bone Loss,” and “Diabetic Retinopathy.” Both MeSH and entry terms were correctly adapted according to the syntax rules for each database and gray literature, as shown in Supplementary Table 2, using Boolean operators (OR, AND) to combine terms. To find additional citations, a manual search was performed in the references of the included studies, and a search alert was activated in each database to received notifications when there were new articles that matched our search query.

All the citations found on databases and by hand search were entered into reference management software (EndNote®, version X7, Thomson Reuters, Philadelphia, USA). The identified duplicates were manually and automatically excluded.

Titles, abstracts, and full text were independently analyzed for eligibility by two review authors (MA and GM), and in case of disagreement between them, a third reviewer was consulted to make the final decision (RL).

### Data Extraction and Risk of Bias Assessment

To summarize the main findings, the following data were extracted from the included articles: authors and year, study design, characteristics of the sample (size, age, location, and study group); evaluation method (from periodontitis, diabetes mellitus, and diabetic retinopathy diagnosis), statistical analysis, results (study group, control group, and the *P*-value), and outcomes.

A score for quality synthesis modified from the Newcastle–Ottawa scale (12) was used to assess the appropriateness of research design, recruitment strategy, response rate, representativeness of the sample, objectivity/reliability of outcome determination, power calculation provided, and appropriate statistical analyses. Studies were classified into three groups of different qualities (good, fair, and poor) based on the following parameters: good quality—three or four stars in the selection domain AND one or two stars in the comparability domain AND two or three stars in the outcome/exposure domain; fair quality—two stars in the selection domain AND one or two stars in the comparability domain AND two or three stars in the outcome/exposure domain; poor quality—zero or one star in the selection domain OR zero star in the comparability domain OR zero or one star in the outcome/exposure domain (Supplementary Text 3). Score disagreements were resolved by consensus with assistance from a third reviewer.

### Assessing the Quality of the Body of Evidence (GRADE)

The evidence of all studies taken together was evaluated using the Grading of Recommendations Assessment, Development, and Evaluation (GRADE) approach (Guideline Development Tool, available online at gradepro.org) (13). In this review, a narrative GRADE was chosen according to the types of the included studies.

In this assessment, the certainty of the evidence was evaluated according to the study design, risk of bias, inconsistency, indirectness, and imprecision parameters, in order to be categorized into one of four ratings—high, moderate, low, and very low—that reflect the extent to which the review authors are confident that an estimate of the effect for a specific outcome is correct (14).

However, observational studies are initially rated as “low,” and then other issues within the magnitude of the effect, inconsistency, indirectness, imprecision, and counteracting plausible residual bias or confounding could be used to downgrade the evidence (15).

## Results

### Characteristics of the Included Studies

**Figure 1:**
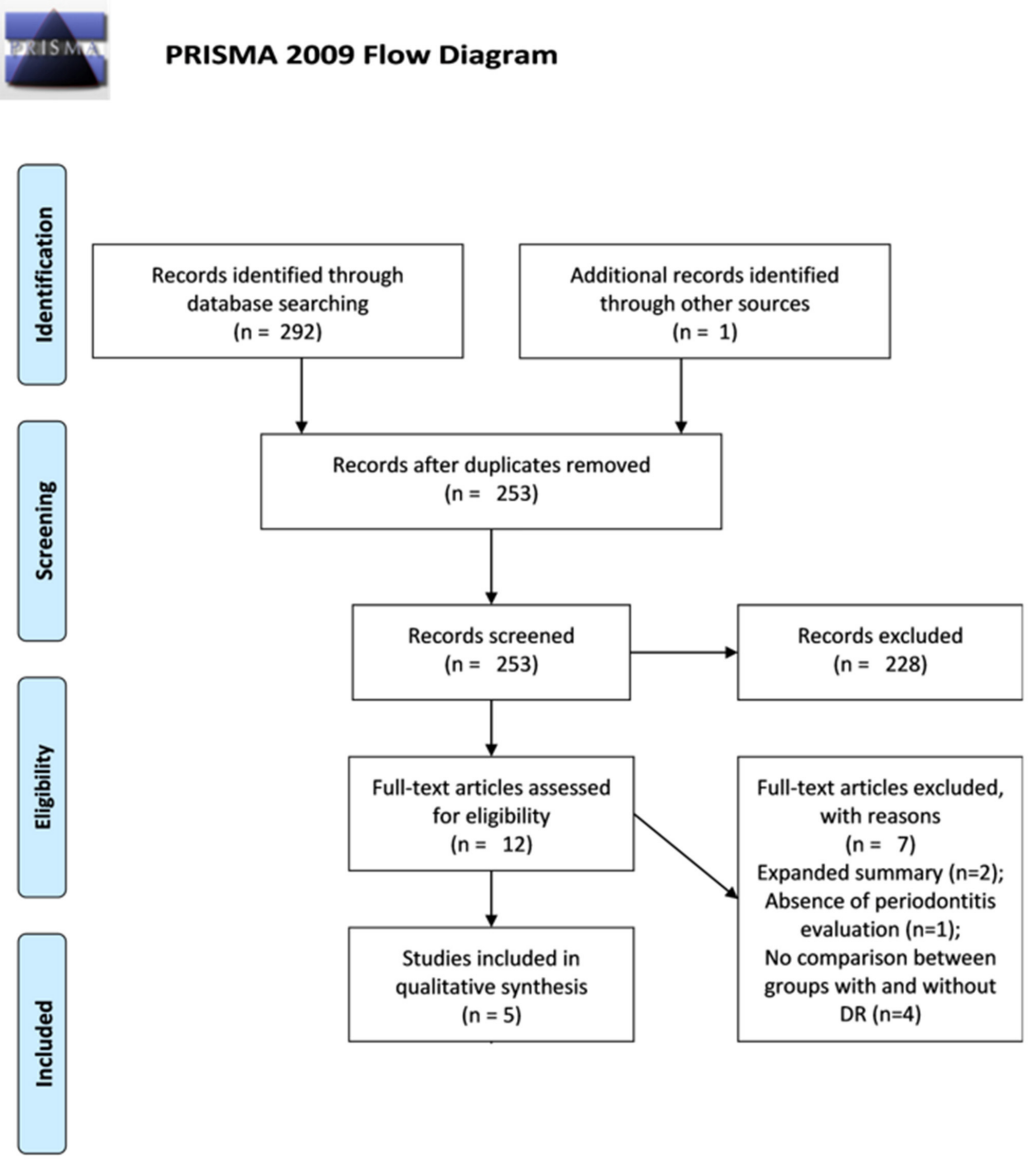
Preferred reporting items for systematic review and meta-analysis (PRISMA) flow diagram. The flow diagram depicts the flow of information through the different stages of this systematic review: the number of records identified, included and excluded, and the reasons for exclusions.

The search strategy and hand search identified a total of 293 records. After removing the overlaps, 253 records were assessed by title and abstract, and out of them, 12 were eligible for full-text reading. After the full-text analysis based on the aforementioned eligibility criteria, seven articles were excluded because they did not meet the inclusion criteria, and the remaining five were considered for qualitative analysis as shown in Figure 1.

The five studies included were cross-sectional reports, carried out between 1994 and 2018, and covering 1,009 participants. Regarding the association between periodontitis and diabetic retinopathy, in all the included studies was found a significant difference in the prevalence of diabetic retinopathy in diabetic patients with periodontitis compared to those without periodontitis as shown in Table 1.

**Table 1:** Summary of characteristics of the included studies.

**References/country/study design**	**Sample**		**Diabetes**		**Diabetic retinopathy evaluation**	**Periodontal evaluation**	**Statistical analysis**	**Results**
	**Sample size (*n*)**	**Mean age**	**Type (mean time since diagnosis in years)**	**Diagnostic technique**				
Veena et al. (11) India Cs	G1: Mild to moderate non-PDR: 55 G2: Moderate to severe non-PDR: 44 G3: PDR: 52 G4: Control group: 49	–	2	HbA1c test	Eye examination (unspecified)	PI, GI, PPD, and CAL.	ANOVA one-way with Tukey's *post hoc* test; *Student's T*-*test*	A statistically significant correlation between the severity of DR and the severity of periodontitis was found (*p* < 0.001)
Song et al. (16) Korea Cs	Diabetics (1.138) Diabetics with DR: 90 Diabetics without DR: 468	Diabetics with DR: 58.5 ± 1.6 Diabetics without DR: 57 ± 0.7	2 (With RD: 10.9 ± 1.0) (Without RD: 4.7 ± 0.3)	HbA1ctest	Nonmydriatic fundus photography (TRC-NW6S; Topcon, Tokyo, Japan)	CPI	Multiple logistic regression	The risk of DR was positively associated with the presence of periodontitis in non-obese diabetic
Amiri et al. (17) Iran Cs	Diabetics with DR: 84 Diabetics without DR: 129	–	2	HbA1c test	Fundus examination by a trained ophthalmologist	PI, CPI, PPD, and CAL	Multivariable logistic regression	The severity of periodontitis was significantly correlated with the severity of DR (*p* < 0.011)
Ota et al. (18) Japan Cs	Diabetics with PDR: 7 Diabetics with pre-PDR: 9 Diabetics without DR: 7	55.4 ± 11.3	2 (10.3 ± 8.8)	HbA1c test	Eye examination (unspecified)	Number of teeth present, PPD, BOP, and tooth mobility	Chi-square test	The average of patients with severe periodontitis (SD ± 7 mm) was significantly higher in patients with DR
Karjalainen et al. (19) Finland Cs	Diabetics with DR: 11 Diabetics without DR: 4	Male: 30.9 ± 2.7 Female: 29.6 ± 2.1	1	All information was collected from patient records	Direct and indirect ophthalmoscopy. Fundus photographies were taken	PI, PPD, CAL, and BOP	Chi-square test	Differences were significant (*P* = 0.05) between the groups with and without DR. The severity of periodontitis increased with the severity of DR.

*G1, Group 1; G2, Group 2; G3, Group 3; DR, diabetic retinopathy; PDR, proliferative diabetic retinopathy; HbA1c, glycated hemoglobin; Cs, cross-sectional study; PI, plaque index; GI, gingival index; PPD, probing pocket depth; CAL, clinical attachment level; BOP, bleeding of probing; CPI, community periodontal index*.

For the assessment of diabetes mellitus, the glycated hemoglobin (HbA1c) test was used on the participants to determine the clinical diagnosis. Only one study was based on information registered in the patient records, considering HbA1c values (up to 5 years retrospectively), duration of diabetes, and details of diabetic complications (19). The evaluation of retinopathy was performed using an ophthalmic examination. Two studies did not specify the type of test used (11, 18), while the others cited ophthalmoscopy as the exam of choice.

The status of the periodontium analysis was carried out using validated clinical parameters, such as plaque index (PI), gingival index (GI), probing pocket depth (PPD), bleeding of probing (BOP), and clinical attachment level (CAL). One study used only the community periodontal index (CPI) (16). PPD was the most used, observed in four studies (11, 17–19), followed by the PI (11, 17, 19) and CAL (11, 17, 18), both used in three studies. BOP was analyzed in two studies (18, 19), as well as the CPI (16, 17).

The relationship between periodontitis and retinopathy was investigated based on the association provided by diagnostic parameters of periodontitis and the occurrence of diabetic retinopathy. Odds ratio analysis between diabetic retinopathy and periodontitis was performed by Song et al. (odds ratio adjusted for HbA1c level, duration of diabetes mellitus, and white blood cell count−2.206 [1.114–4.366]; *p* = 0.0232). A positive relationship between severity of diabetic retinopathy and severity of periodontitis was found by four authors (11, 17–19).

### Quality and Risk of Bias Assessments

Among the five studies included, two were classified as good quality, one as fair quality (11), and two studies as poor quality (18, 19). Most problems were related to domains case definition, sample selection, and statistical analysis of outcomes. Case description, matching problems between groups, nonrepresentative samples, and lack of relevant confounder control in some of the studies diminished the overall quality accordingly. The Newcastle–Ottawa assessment scale adapted for cross-sectional studies. The results of this quality assessment are shown in Table 2.

**Table 2:** Assessment of quality and risk of bias by the Newcastle–Ottawa scale.

**Cross sectional**	**Amiri et al. (17)**	**Karjalainen et al. (19)**	**Song et al. (16)**	**Veena et al. (11)**	**Ota et al. (18)**
**Selection (5 stars max)**					
1) Is the case definition adequate?	*	–	*	–	–
2) Sample	*	–	*	–	–
3) Non-respondents	–	–	*	*	–
4) Ascertainment of the exposure (risk factor)	**	*	**	*	*
**Comparability (2 stars max)**					
1) The subjects in different outcome groups are comparable, based on the study design or analysis. Confounding factors are controlled	*	*	**	*	–
**Outcome (3 stars max)**					
1) Ascertainment of outcome	**	**	**	**	**
2) Statistical test	*	*	*	-	*
Overall	Good Quality	Poor Quality	Good Quality	Fair Quality	Poor Quality

Domain evaluation score: one star: “*”; two stars: “**”; three stars: “***”. A “star system” has been developed to evaluate studies on three broad perspectives: the selection of the study groups; the comparability of the groups; and the ascertainment of either the exposure or outcome of interest for case-control, cross-sectional, or cohort studies, respectively.

### Quality of the Body of Evidence (GRADE)

A narrative GRADE approach was applied to assess the quality of evidence across the three studies judged to be fair and good quality in the qualitative analysis. Low quality was observed among the outcomes in the overall analysis, in which the flaws presented in the risk of bias and imprecision of analysis because they are observational studies were directly associated with the downgrade of the evidence as shown in Table 3.

**Table 3:** Quality of the body of evidence by a GRADE narrative assessment.

**Certainty assessment**							**Impact**	**Certainty**	**Importance**
**No. of studies**	**Study design**	**Risk of bias**	**Inconsistency**	**Indirectness**	**Imprecision**	**Other considerations**			
**Association between periodontitis and diabetic retinopathy**									
3 ^(11, 16, 17)^	Observational studies	Not serious^a^	Not serious	Not serious	Serious^b^	All plausible residual confounding would reduce the demonstrated effect	In three studies, the association between DR and periodontitis in diabetic patients was evaluated. In all studies, there was a statistically significant prevalence of RD in patients with periodontitis and diabetes. However, problems related to reduced sample sizes impair precision analysis of these association	⊕⊕○○ LOW	IMPORTANT

*CI, confidence interval*.

a *Three studies analyzed groups without confounder adjustments*.

b *Small sample sizes for imprecision evaluation*.

⊕○○○*Very low;* ⊕⊕○○*Low;* ⊕⊕○○*Moderate;* ⊕⊕⊕○*High;* ⊕⊕⊕⊕*Very high. Quality of evidence may vary to high (high confidence on the estimated effect), to moderate (moderate confidence of the effect), to low or very low (little confidence on the estimated effect, the true effect is likely to be substantially different from the estimate of effect)*.

## Discussion

This systematic review proposed to investigate the association between diabetic retinopathy, a microvascular complication of diabetes mellitus, and periodontal disease, in patients diagnosed with diabetes mellitus. Five studies met the eligibility criteria and were included in the review. In all the included studies was found a statistically significant association between the prevalence of diabetic retinopathy and periodontitis. However, only two studies were classified as good according to the quality assessment. Furthermore, the certainty of the evidence of all the outcomes evaluated was classified as very low by the GRADE tool.

A systematic review is a type of study in which the purpose is to summarize the results of available studies about medical questions, providing a high level of scientific evidence (19) make clinical decisions in therapeutics and health conducts, through a step by step process (20). Thus, all steps of this review were made according to the PRISMA standards and following a PECO acronym suggested by guidelines for systematic reviews and meta-analysis (21) and the focus question was: Is there any association between diabetic retinopathy and periodontitis?

Diabetes and periodontitis have been considered among the most prevalent human disorders, and frequently, they are presented simultaneously in many patients (22). Thus, the signs and symptoms of periodontitis have been recognized as the sixth complications of diabetes mellitus (11). Periodontitis is a chronic inflammatory condition that produces increased circulating cytokines and inflammatory mediators—such as IL-1β, IL-6, C-reactive protein, TNF-α, PGE2, and receptor activator of nuclear factor κB ligand (RANKL) as an autoimmune response to the chronic periodontal infection (8, 23). The chronic bacterial challenge in periodontitis is a persistent source of inflammatory mediators (8) and may trigger an impaired glucose homeostasis/increased insulin resistance. It has been theorized that proinflammatory cytokines enter the bloodstream leading to exacerbation of diabetes and increasing the risk of its complications. Conversely, the elevated levels of the proinflammatory cytokines in diabetic patients may reach the gum and exacerbate existing periodontal disease, thus proposing a bidirectional relationship between periodontitis and diabetes mellitus (24). Diabetic retinopathy is a common microvascular complication of chronic diabetes and is considered the main cause of visual loss worldwide (25). Prolonged exposure to high blood glucose due to lack of long-term diabetes control constitutes a risk factor for diabetic retinopathy (1, 2, 4). However, there are patients with diabetic retinopathy that do not present high blood glucose and patients with long-term diabetes that do not progress retinopathy (5). The main reasons for this decline in visual activity are the blood–retinal barrier disruption with consecutive diabetic macular edema and retinal angiogenesis, but the exact mechanism that promotes this vascular disruption is not completely understood (5).

An increased risk for poor glycemic control (HbA1c > 9%, 75 nmol/ml) has been associated with patients with moderate to severe periodontitis and edentulous patients (≥ 2 years follow-up). Poor glycemic control resulting from periodontitis may increase the risk of the onset of other systemic complications, such as macroalbuminuria (2–2.5 higher in patients with moderate to severe periodontitis), end-stage kidney disease (2.3–3.5 times higher in patients with moderate to severe periodontitis), and an increased risk for cardiorenal mortality (3.2 times more likely for patients with moderate to severe periodontitis) (26–28).

Several biochemical mechanisms have been proposed to modulate the pathogenesis of diabetic retinopathy; among the different pathways implicated are oxidative stress and inflammation (5). There is growing evidence that inflammation—in response to hyperglycemia and other stresses, plays an important role in the development of this diabetes complication (2, 5, 29). The activity of IL-1β, IL-6, TNF-α, and C-reactive protein mediators in the recruitment of polymorphonuclear cells increases the production of reactive oxygen species (ROS) (8, 9). The ROS alter vascular permeability by increasing the ONOO– peroxynitrites and result in endothelial damage, thus exacerbating the vascular changes promoted by diabetes (30). In this regard, the same biomarkers related to oxidative stress were found in patients with periodontitis, showing oxidative stress as a biochemical event associated with the destruction of periodontal tissue (31).

Another mechanism proposed is based on the fact that periodontitis can also lead to atherosclerosis resulting in hypoxia in the retina and causing proliferation of new fragile and leaky vessels and eventually retinal detachment (11, 29). Systemic concentrations of C-reactive protein, TNF-α, IL-1b, and IL-6 are associated with a reduction in proteins responsible for the balance of triglyceride-derived lipids and an increase in adipose tissue macrophages (ATMs) (32). With the increase in ATMs, a greater restriction on lipases occurs, resulting in a higher concentration of lipids in the bloodstream causing ectopic fat deposits, mainly in the endothelium (32). In this way, the related endothelium can trigger hypoxia in the retinal region possibly resulting in diabetic retinopathy (9).

Otherwise, diabetes can also be understood as a risk factor for periodontitis. Previous studies suggest that patients with diabetes show increased susceptibility to the progress of periodontitis (17, 33). During the pathogenesis process of periodontitis, damage to dental supporting tissues (gingiva, cementum, periodontal ligament, and alveolar bone) occurs due to the imbalance of the host's response to dental plaque. A complex interaction between inflammatory mediators results in increased activity of metalloproteinases (MMPs; particularly MMP-8, MMP-9, and MMP-13), reactive oxygen species, and increased bone resorption (8). The increase in IL-1β, IL-6, and TNF-α occurs due to the lipopolysaccharides present in the biofilm; nonetheless, the increase is accentuated when there is an accumulation of AGEs—a common event in diabetic patients (8). Thus, both diabetes and periodontitis might influence each other in a synergistic relationship (34, 35).

In this systematic review, the five observational studies that met our eligibility criteria reported a significant relationship between the prevalence of diabetic retinopathy and periodontal disease in their statistical analyses (11, 16–19). All of the included studies compared the presence of diabetic retinopathy in diabetic patients with and without periodontitis using similar techniques to evaluate periodontal and retina status. To measure the status of the periodontium, the studies used validated clinical parameters. Periodontitis is characterized by the presence of PPD >4 mm, BOP >10% of sites, and CAL >3 mm (35, 36). Regarding the diagnosis of diabetic retinopathy, out of the five studies, two did not specify the type of eye examination used (11, 18), and the remaining four performed fundus photography (16, 17, 19).

Furthermore, two studies included in this review evaluated proliferative diabetic retinopathy (PDR) and severe periodontitis and found a significant correlation between them (11, 18). In this regard, several studies support a relationship between some persistent systemic inflammation and angiogenesis, and this interplay might be also critical for PDR initiation and progression (37, 38). The link between them is possibly related to inflammasome NLRP3 (nucleotide-binding domain and leucine-rich repeat receptor containing a pyrin domain 3), a protein responsible for several intracellular signaling events, present in periodontitis pathogenesis and also present in the retina of patients with PDR, especially when it is partially or fully detached (23, 29, 39).

## Conclusions

Although all evidence gathered in this review suggests an association between the severity of diabetic retinopathy and the severity of periodontitis, the quality of the body of the evidence was judge to be *low* by the GRADE assessment, and this relationship cannot be reliably established. Despite only two studies were judge to be of poor quality by the Newcastle–Ottawa scale, all of them did not control all the confounding factors for periodontitis and diabetic retinopathy, and the sample sizes were not large enough to provide a strong association. Further studies with larger sample sizes, appropriate adjustment of measured confounders, as well as a prospective analysis of both conditions ought to be conducted to clarify whether periodontitis is a risk factor in the progression of diabetic retinopathy or a coincidental finding. However, considering the recurrence of this association, dentists must consider the risk of diabetic retinopathy while managing diabetic patients, especially with periodontitis, and refer to an ophthalmologist for an eye examination.

## Data Availability Statement

The original contributions presented in the study are included in the article/Supplementary Material, further inquiries can be directed to the corresponding author/s.

## Author Contributions

MP, NF, and RL conceptualized the study. MP, GM, and RF were in charge of the methodology. RL, LM, NF, and MS validated the study. LM and RL were in charge of the formal analysis. MP prepared and wrote the original draft. RL, MS, and RF wrote, reviewed, and edited the manuscript. All authors have read and agreed to the published version of the manuscript.

## Conflict of Interest

The authors declare that the research was conducted in the absence of any commercial or financial relationships that could be construed as a potential conflict of interest.
